# Associations of body image with depressive symptoms and PTG among breast cancer patients: The mediating role of social support

**DOI:** 10.3389/fpsyg.2022.953306

**Published:** 2022-10-14

**Authors:** Mengyao Li

**Affiliations:** Department of Social Medicine, School of Health Management, China Medical University, Shenyang, China

**Keywords:** posttraumatic growth, depressive symptoms, social support, body image, breast cancer

## Abstract

**Background:**

Cancer diagnosis and treatment usually trigger positive and negative psychological health outcomes. Social support is a coping resource for psychological health outcomes. However, little research is available on the relationships between social support, body image, and overall psychological health outcomes in breast cancer (BC) patients. This study aimed to estimate the prevalence of depressive symptoms and post-traumatic growth (PTG) and examine the mediating roles of social support between body image and depressive symptoms and PTG among BC patients, respectively.

**Methods:**

A cross-sectional study was conducted in the Northeast China from December 2015 to August 2017. All the participants were diagnosed with BC and underwent surgery. This study was conducted with 405 BC patients from the First Affiliated Hospital of China Medical University. Participants completed the Center for Epidemiologic Studies Depression scale, Post Traumatic Growth Inventory, Body Image Scale, and Perceived Social Support Scale. The associations of social support, body image with depressive symptoms, and PTG were examined by hierarchical linear regression analysis. Asymptotic and resampling strategies were used to explore the mediating role of social support.

**Results:**

The prevalence of depressive symptoms was 88.1%, and 67.2% of the patients had moderate-high PTG, 52.84% of the patients had body concerns, and 264 (65.19%) patients had high-level social support. Body image was positively associated with depressive symptoms (β = 0.445, *P* < 0.001) and social support was negatively associated with depressive symptoms (β = −0.219, *P* < 0.001). Body image was negatively associated with PTG (β = −0.095, *P* = 0.023), whereas social support was positively associated with PTG (β = 0.533, *P* < 0.001). Social support significantly mediated the associations among body image, depressive symptoms (effect size = 0.057), and PTG (effect size = −0.304), respectively.

**Conclusions:**

Social support played mediating role in the relationships between body image and depressive symptoms and PTG. The interventions based on social support and body image should be included in psychological health prevention.

## Background

Breast cancer (BC) became the leading diagnosed cancer worldwide in 2020 (Cao et al., 2021). The mortality of BC is increased rapidly, and female BC patients in China accounted for approximately 18% of the global BC deaths (Cao et al., 2021). Characteristics such as the long process of exposure to the disease and the untreatable nature will seriously impact the mental health of BC patients.

Both the diagnosis of BC and its treatment can cause serious psychological burdens on patients (Miller et al., 2016). Depression is one of the most common psychiatric symptoms in patients with BC. According to the literature review, the prevalence of mild or moderate depression was between 8.1 and 89.2%, and the prevalence of major depression was between 1.8 and 69.4% (Pilevarzadeh et al., 2019). Depressive symptoms can persist for at least 2 years after diagnosis (Avis et al., 2015). Depression can worsen the feeling of discomfort and further result in poorer outcomes (Eskelinen et al., 2017). However, depression in BC patients is often overlooked and undertreated.

BC diagnosis can not only trigger negative reactions but also cause positive psychological outcomes (Romeo et al., 2020). According to the model of Tedeschi and Calhoun (2004), posttraumatic growth (PTG) is a beneficial outcome of struggle with a traumatic event. PTG is defined as positive cognitive and affective changes experienced, as a result of the struggle with a traumatic event (Tedeschi and Calhoun, 2004). These changes occur across three broad domains, life perspective, interpersonal relationship, and self-perception. Through these changes, a new life structure is created, which leads to positive health outcomes, such as adaptive responses to cancer, spiritual wellbeing, positive affect, and positive lifestyle changes (Costa and Pakenham, 2012). Previous studies usually focus on a single psychological outcome (negative or positive reaction) among Chinese BC patients, but studies exploring psychological outcomes from both positive and negative aspects among Chinese BC patients are rather limited.

BC patients typically face several treatment options, and side effects of these treatments can cause bodily changes (Fetaini et al., 2020). Bodily changes include breast removal, scarring, hair loss, or other appearance alterations (Fingeret et al., 2014; Trindade et al., 2018; Alhusban, 2019). Body image difficulties can affect psychological health and increase the risk of mortality considerably (Alhusban, 2019). Poorer body image has been found to link to overall psychological distress in BC patients (Trindade et al., 2018). Nevertheless, most studies focus on associations between body image and negative psychological outcomes till now (Trindade et al., 2018; Alhusban, 2019), and neglect the influence of body image on PTG. Body consciousness is part of a social or culturally influenced reaction, that can impede psychological adjustment after BC treatments (Trindade et al., 2018). Disturbances in body image have been considered a barrier to positive factors (Izydorczyk et al., 2018). Whereas, Hefferon et al. (2010) reviewed that the body was a vital component of the process and outcomes of PTG, according to an open-ended interview for 10 BC survivors. Further investigation is needed to explore the relationship between body image and psychological outcomes, especially positive outcomes.

Regarding social resources, individuals are willing to seek social support when they require information or assistance to respond to or cope with a certain danger (Jung, 2010). Cancer experiences can lead individuals to experience a sense of personal inadequacy (Biglia et al., 2012), so having strong perceived social support is important to a better cancer adjustment (Zamanian et al., 2021). Studies have confirmed that social support helps promote psychological health outcomes (Cao et al., 2018) and relieve psychological distress among cancer patients (Zamanian et al., 2021).

## Conceptual framework

Social support is usually considered a coping strategy and mediating construct in dealing with cancer trajectory. This mediation approach was informed by the Response Shift Framework (Sprangers and Schwartz, 1999). Response shift is a potential explanation when an individual is experiencing a serious health event or chronic condition. Response shift refers to a change in the meaning of one's self-evaluation of a target construct as a result of changes in the respondent's internal standards of measurement, the respondent's values, or as a redefinition of the target construct (Sprangers and Schwartz, 1999). A cancer diagnosis can lead to changes in body image, which may result in a response shift, and subsequently changes in psychological outcomes. Mechanisms will refer to coping strategies (social support) to accommodate the catalyst (cancer diagnosis) (Manne et al., 2018; Kugbey et al., 2020). Body image had been predicted to be associated with social support (Spatuzzi et al., 2016; Katapodi et al., 2018). However, the relationships between social support, body image, and psychological outcomes (depression and PTG) remain unclear among BC patients.

In light of the above concerns, the purpose of the present study is to verify the following two assumptions among Chinese BC patients: (1) body image and social support are associated with depressive symptoms and PTG and (2) social support mediates the associations between body image and depressive symptoms, PTG, respectively.

## Methods

### Ethical approval

The study design was approved by the Committee on Human Experimentation of China Medical University. Informed consent was signed by each participant. The privacy of patients was protected throughout the entire research.

### Participants and procedures

Cross-sectional research was conducted in Liaoning Province, from December 2015 to August 2017. All participants were from the Department of Breast Surgery, First Affiliated Hospital of China Medical University. Inclusion criteria in this study were as follows: (1) 18 years old or above, (2) with pathological diagnosis of breast cancer, (3) underwent surgery, and (4) aware of their cancer diagnosis. Exclusion criteria included that patients had (1) intellectual impairments, (2) a history of psychiatric problems before cancer diagnosis, and (3) other active tumors. Self-administered questionnaires were distributed to the patients after obtaining their informed consent. There were strict quality control measures to avoid possible bias. Each patient has distributed the questionnaire to complete in a private place within 1 week after surgery. Of the 458 who met the inclusion criteria, 53 patients were excluded because they declined to participate or the missing values exceeded 30% of the questionnaire. The final response rate was 88.43%.

### Study measures

Four self-report instruments were employed in this study, including the Center for Epidemiologic Studies Depression (CES-D) scale, Post Traumatic Growth Inventory (PTGI), Body Image Scale (BIS), and Perceived Social Support Scale (PSSS). The background schedules were used to collect demographic factors and clinical information.

### Measurement of depressive symptoms

Depressive symptoms were measured with the Center for Epidemiologic Studies Depression (CES-D) scale (Radloff, 1977). The CES-D scale comprises 20 items, for example, “I felt sad.” Each item had four answers ranging from 0 (rarely) to 3 (most). The score ranges from 0 to 60. The presence of depressive symptoms was defined as a CES-D score ≥16. The Chinese version had been used in previous study (Yang et al., 2015). The Cronbach's α for CES-D was 0.855.

### Measurement of PTG

PTG was assessed using the 21-item Post Traumatic Growth Inventory (PTGI) (Tedeschi and Calhoun, 1996). The example question includes “I established a new path for my life.” Each item has six responses ranging from 0 “I did not experience this change as a result of my cancer” to 5 “I experienced this change to a very great degree as a result of my cancer,” with higher scores indicating greater PTG. Mean item scores <3 are considered as low PTG (Jansen et al., 2011), and scores ≥3 are considered as moderate to high PTG. In this present study, the cut-off score was used only to describe the level of PTG, and the total PTGI score was used for the statistical analysis. The Chinese version had been widely used and demonstrated adequate reliability and validity (Wang et al., 2018). The Cronbach's α for PTGI was 0.940.

### Measurement of body image

Body image was measured with Body Image Scale (BIS), including 10 items (Hopwood et al., 2001). Example question includes “Have you been feeling self-conscious about your appearance.” Each item had four responses ranging from 0 (not at all) to 3 (very much). The total score ranges from 0 to 30, and higher scores represented increasing symptoms/distress. The Chinese version had been widely used and had adequate reliability and validity (Chen et al., 2015). The Cronbach's α for BIS was 0.900. A sum score of 10 or greater is an indicator of body image concerns (Chopra et al., 2021).

### Measurement of social support

Social support was assessed using the 12-item version Perceived Social Support Scale (PSSS) (Zimet et al., 1988), such as “I can talk about my problems with my family.” The item is a 7-point rating ranging from 1 “very strongly disagree” to 7 “very strongly agree.” Total scores range from 12 to 84, and the higher total score reflects better social support. Participants with scores of 61–84 indicated high-level perceived social support, 37–60 indicated medium-level perceived social support, and 12–36 indicated low-level perceived social support (Wang et al., 2022). Chinese version had been used in previous studies, and the reliability and validity had been repeatedly confirmed (Huang et al., 1996). The Cronbach's α for PSSS was 0.950.

### Demographic and clinical variables

Demographic factors included age, marital status, education, monthly income, residence, and physical activity. Age was categorized into three groups: “ ≤39 years old,” “40-49 years old,” and “≥50 years old.” Marital status was categorized as “single/separated/divorced/widowed” and “married/cohabitated.” Education was categorized as “primary/middle school,” “high school,” and “junior college or above.” Monthly income was divided into three groups: “ <3,000 yuan,” “3,000–5,000 yuan,” and “>5,000 yuan.” Residence was categorized as “rural” and “urban.” Physical activities were divided into two groups: “no” and “yes.”

Clinical variables included type of diagnostic, chemotherapy, radiotherapy, metastasis, and cancer stages. The type of diagnostic was divided into “recurrence” and “new.” Chemotherapy, radiotherapy, and metastasis were divided into “no” and “yes” two groups. Cancer stages were categorized into three groups: “I,” “II,” and “III.”

### Statistical analysis

Statistical analysis was executed by SPSS 22.0 software. Frequency and percent were used as descriptive statistics for demographic and clinical variables. The suitability of the data for normal distribution was evaluated by the Kolmogorov–Smirnov test. Since the depressive symptoms and PTG conformed to normal distributions, demographic and clinical variables were examined for inclusion as covariates by using an independent sample t-test or one-way analysis of variance (ANOVA). Correlations among normally distributed variables were examined using Pearson's correlation, and correlations among not normally distributed variables were examined using Spearman's correlation. We imputed missing values by the mean values for each parameter. Hierarchical linear regression analyses were performed to examine the associations of body image and social support with depressive symptoms and PTG, respectively. Body image was entered as an independent variable, with PTG and depressive symptoms as outcomes, social support as a mediator, and demographic or clinical variables as control variables. A total of two models were developed. Based on the results of univariate analysis, education, physical activity, chemotherapy, and monthly income were entered into step 1 for the model of depressive symptoms; and marital status and type of diagnostic were entered into step 1 for the model of PTG. Body image was entered into step 2, and Social support was entered into step 3. Variance inflation factor (VIF) was calculated to identify multi-collinearity. Asymptotic and resampling strategies were used to examine social support as a potential mediator in the association between body image and PTG and depressive symptoms based on 5,000 bootstrap samples (Preacher and Hayes, 2008). A bias-corrected and accelerated 95% confidence interval (BCa 95% CI) was estimated for each mediation (a × b product), and a BCa of 95% CI excluding 0 indicated a significant mediating role. A two-tailed *P* < 0.05 was viewed as statistically significant.

## Results

### Participant characteristics

Of the 405 breast cancer patients, the mean (SD) age was 49.80 (9.66) years (range 27–75). Most patients (85.2%) were married or cohabited. In total, 164 (40.5%) participants had a primary or middle school degree. According to our results, 305 (75.3%) participants came from urban areas. Approximately 87.9% were newly diagnosed and 227 (56.0%) were diagnosed at cancer stage II. The prevalence of depressive symptoms among BC patients was 88.1%. In view of depressive symptoms, education (*F* = 6.211, *P* = 0.002), monthly income (*F* = 6.466, *P* = 0.002), physical activity (*t* = 2.625, *P* = 0.009), and chemotherapy (*t* = 2.471, *P* = 0.014) were all significantly related with depressive symptoms. Additionally, the mean (SD) PTGI item score was 3.29 (0.80), and 67.2% reported moderate to high PTG (mean item score ≥ 3). Married/cohabited participants reported higher PTGI scores (*t* = −2.263, *P* = 0.027) than those single/divorced/widowed/separated ones. New cases had higher PTGI scores (*t* = −3.682, *P* < 0.001) than those with cancer recurrence. Results were shown in Table 1.

**Table 1:** Univariate associations of demographic and clinical characteristics with depressive symptoms and PTG (*N* = 405).

**Variables**	***N* (%)**	**Depressive symptoms**	***t*/F**	***P***	**PTG**	***t*/F**	***P***
		**(Mean ± SD)**			**(Mean ± SD)**		
**Age**							
≤39	60 (14.8)	26.70 ± 8.47	1.155	0.316	67.89 ± 15.20	0.167	0.846
40–49	142 (35.1)	24.99 ± 8.22			69.37 ± 16.22		
≥50	203 (50.1)	24.96 ± 7.99			69.08 ± 17.82		
**Marital status**							
Single/divorced/widowed/separated	60 (14.8)	25.90 ± 8.34	0.694	0.488	63.59 ± 20.72	−2.263	0.027
Married/cohabited	345 (85.2)	25.11 ± 8.12			69.95 ± 15.95		
**Education**							
Primary/Middle school	164 (40.5)	26.89 ± 7.65	6.211	0.002	68.29 ± 17.08	2.649	0.072
High school	121 (29.9)	24.52 ± 7.96			67.14 ± 16.37		
Junior college or above	120 (29.6)	23.67 ± 8.63			71.87 ± 16.84		
**Monthly income (yuan)**							
<3,000	249 (61.5)	26.26 ± 7.89	6.466	0.002	67.91 ± 17.16	2.367	0.095
3,001–5,000	107 (26.4)	24.26 ± 7.95			69.47 ± 17.20		
≥5,001	49 (12.1)	22.13 ± 8.94			73.56 ± 13.85		
**Residence**							
Rural	100 (24.7)	25.16 ± 8.43	−0.093	0.926	70.54 ± 16.41	1.046	0.296
Urban	305 (75.3)	25.25 ± 8.06			68.50 ± 17.01		
**Physical activity**							
No	156 (38.5)	26.56 ± 7.53	2.625	0.009	67.08 ± 19.12	−1.728	0.085
Yes	249 (61.5)	24.39 ± 8.42			70.21 ± 15.21		
**Type of diagnostic**							
Recurrence	49 (12.1)	25.13 ± 8.53	−0.092	0.927	60.81 ± 16.60	−3.682	<0.001
New	356 (87.9)	25.24 ± 8.10			70.13 ± 16.61		
**Chemotherapy**							
No	32 (7.9)	28.62 ± 7.61	2.471	0.014	67.96 ± 19.50	−0.366	0.715
Yes	373 (92.1)	24.94 ± 8.13			69.10 ± 16.65		
**Radiotherapy**							
No	330 (81.5)	25.39 ± 8.11	0.859	0.391	69.31 ± 16.26	0.767	0.444
Yes	75 (18.5)	24.50 ± 8.30			67.66 ± 19.38		
**Metastasis**							
No	351 (86.7)	25.09 ± 8.32	−0.875	0.382	69.64 ± 16.93	1.931	0.054
Yes	54 (13.3)	26.13 ± 6.92			64.89 ± 16.02		
**Cancer stage**							
I	118 (29.1)	25.59 ± 7.57	0.242	0.785	71.18 ± 17.13	2.933	0.054
II	227 (56.0)	24.98 ± 8.78			67.22 ± 16.68		
III	60 (14.8)	25.44 ± 6.68			71.49 ± 16.51		

SD, standard deviation; PTG, posttraumatic growth.

### Correlations among continuous variables

Correlations among study variables were presented in Table 2. The mean score (SD) for depressive symptoms was 25.23 (8.15), the mean score (SD) for PTGI was 69.01 (16.87), the mean score (SD) for body image was 10.41 (6.52), and the mean score (SD) for social support was 64.74 (13.91). Only 191 (47.16%) patients indicated that they had low body concerns and 264 (65.19%) patients had high-level social support. Social support was negatively correlated with depressive symptoms (*r* = −0.346, *P* < 0.01) and positively correlated with PTG (*r* = 0.610, *P* < 0.01), respectively. Body image was positively correlated with depressive symptoms (*r* = 0.515, *P* < 0.01) and negatively correlated with PTG (*r* = −0.230, *P* < 0.01).

**Table 2:** Correlations among study variables.

**Variable**	**Mean (SD)**	**1**	**2**	**3**	**4**
1. Depressive symptoms	25.23 (8.15)	1			
2. PTG	69.01 (16.87)	−0.231^a**^	1		
3. Social support	64.74 (13.91)	−0.346^b**^	0.610 ^b**^	1	
4. Body image	10.41 (6.52)	0.515 ^b**^	−0.230 ^b**^	−0.237 ^b**^	1

** *P* < 0.01.

a Pearson's correlation analysis;

b Spearman's correlation analysis.

PTG, posttraumatic growth; SD, standard deviation.

### Associations of social support and body image with depressive symptoms and PTG

The results of hierarchical linear regression analyses on the associations of social support and body image with depressive symptoms and PTG were presented in Table 3. After adjusting for covariates, body image was positively associated with depressive symptoms (β = 0.490, *P* < 0.001) in step 2. In step 3, social support was negatively associated with depressive symptoms (β = −0.219, *P* < 0.001). Body image accounted for 23.5% of the variance of depressive symptoms, and social support accounted for an additional 4.5% of the variance of depressive symptoms. The tolerance (0.613–0.993) and VIF (1.007–1.631) suggest that there was no multi-collinearity issue in the model.

**Table 3:** Associations of body image and social support with depressive symptoms and PTG.

**Variables**		**Step 1**		**Step 2**		**Step 3**	
		**β**	***p***	**β**	***p***	**β**	***p***
**Depressive symptoms**	Covariates						
	Edu1	−0.114	0.036	−0.105	0.027	−0.090	0.049
	Edu2	−0.101	0.101	−0.090	0.091	−0.076	0.142
	Physical activity	−0.090	0.068	−0.087	0.042	−0.078	0.059
	Chemotherapy	−0.130	0.008	−0.113	0.008	−0.107	0.009
	Monthly income1	−0.130	0.021	−0.071	0.144	−0.052	0.274
	Monthly income2	−0.065	0.232	−0.016	0.734	−0.022	0.631
	Correlates						
	Body image			0.490	<0.001	0.445	<0.001
	Mediating role						
	Social support					−0.219	<0.001
	*F*	4.959	<0.001	24.846	<0.001	26.589	<0.001
	*R^2^*	0.07		0.305		0.349	
	Adjusted *R^2^*	0.056		0.307		0.337	
	*R^2^*-changes	0.07		0.235		0.045	
**PTG**	Covariates						
	Marital status	0.117	0.017	0.099	0.041	0.040	0.327
	Type of diagnostic	0.169	0.001	0.182	<0.001	0.115	0.005
	Correlates						
	Body image			−0.212	<0.001	−0.095	0.023
	Mediating role						
	Social support					0.533	<0.001
	*F*	9.713	<0.001	13.306	<0.001	54.390	<0.001
	*R^2^*	0.046		0.091		0.352	
	Adjusted *R^2^*	0.041		0.084		0.346	
	*R^2^*-changes	0.046		0.044		0.262	

PTG, posttraumatic growth; Edu1, “High school” vs. “Primary/Middle school”; Edu2, “Junior college or above” vs. “Primary/Middle school”; Monthly Income1, “3,001–5,000” vs. “ <3,000”; Monthly Income2, “≥5,001” vs. “ <3,000.”

According to the model of PTG, body image was negatively associated with PTG (β = −0.212, *P* < 0.001) in step 2. In step 3, social support was positively associated with PTG (β = 0.533, *P* < 0.001). Body image accounted for 4.4% of the variance of PTG, and social support accounted for an additional 26.2% of the variance of PTG. The tolerance (0.920–0.990) and VIF (1.010–1.087) suggest that multi-collinearity was not an issue in the estimate.

### Mediating role of social support

Asymptotic and resampling strategies were used to examine the mediating role of social support. As shown in Table 4, body image was negatively associated with social support (*a* = −0.444, *P* < 0.01), and social support (effect size was 0.057, BCa 95% CI: 0.026, 0.104) significantly mediated the association between body image and depressive symptoms.

**Table 4:** Mediating role of social support between body image and depressive symptoms.

**Mediator**	**Variables**	**a**	**b**	**c**	**c^′^**	a*b (BCa95%CI)
Social support	Body image	−0.444**	−0.128**	0.613**	0.556**	0.057 (0.026, 0.104)

** *P* < 0.01.

a = associations of independent variable with the mediator; b = associations of mediator with dependent variable; a^*^b = the product of a and b; c = associations of independent variable with dependent variable; c′ = associations of independent variable with dependent variable after adding the mediator.

BCa 95% CI, bias-corrected and accelerated 95% confidence interval.

Education, physical activity, chemotherapy, and monthly income were added as covariates.

As shown in Table 5, body image was negatively associated with social support (*a* = −0.470, *P* < 0.01), and social support (effect size was −0.304, BCa95% CI: −0.464, −0.174) significantly mediated the association between body image and PTG.

**Table 5:** Mediating role of social support between body image and PTG.

**Mediator**	**Independent variables**	**a**	**b**	**c**	**c^′^**	a*b (BCa95%CI)
Social support	Body image	−0.470**	0.647**	−0.548**	−0.245*	−0.304 (−0.464, −0.174)

* *P* < 0.05;

** *P* < 0.01.

a = associations of independent variable with mediator; b = associations of mediator with dependent variable; a*b = the product of a and b; c = associations of independent variable with dependent variable; c' = associations of independent variable with dependent variable after adding the mediator.

PTG, posttraumatic growth, BCa 95% CI, bias-corrected and accelerated 95% confidence interval.

Marital status and type of diagnostic were added as covariates.

## Discussion

The main objective of this study was to examine the complex relationships between body image and psychological outcomes (depressive symptoms and PTG), and the mediating role of social support in these relationships. The results indicated that depressive symptoms and PTG among BC patients were both at moderate or high levels. Regression analyses showed that each related factor played a specific role in explaining both the negative and positive psychological outcomes. Social support played a positive role in promoting psychological health among Chinese BC patients. In Chinese culture, BC patients were less likely to reveal their cancer diagnosis or treatment, illness-related thoughts, and feelings to outsiders, or to their acquaintances or relatives (Liu et al., 2005). Non-disclosure might be a reason for the high level of depressive symptoms among Chinese BC patients (Lee et al., 2017). Depressive symptoms should be attached great importance in BC patients. In addition, depressive symptoms and PTG might exist at the same time among Chinese BC patients. Measures based on promoting psychological health or promoting the changes from negative to positive psychology were important in psychological intervention.

In our study, most patients (52.84%) had body image concerns. Those with body image concerns were three and a half times more likely to have moderate depression (Chopra et al., 2021). Body image concern was an independent influence factor in damaging psychological health, which might explain the high prevalence of depressive symptoms in this present research. Based on the results, the impact of body image on negative psychology is more severe. These findings were consistent with previous literature (Trindade et al., 2018; Alhusban, 2019). Changes in body image are positively related to depressive symptoms. Increased body image concerns could lead to a higher psychological burden among BC patients (Sherman et al., 2017). Trindade et al. (2018) indicated that a sense of dissatisfaction with an individual's body could cause shame related to cancer, and further aggravate individual's depressive symptoms. In addition, excessive body image disturbance could hinder the development of PTG. A negative body image could cause negative changes in physical wellbeing, emotional wellbeing, social wellbeing, and coping strategies (Alhusban, 2019). So growth process of cancer patients was affected by feelings of loss of control over their bodies (Hefferon et al., 2010). Improving the body image and reconnecting with the body could be a positive stage for reducing depressive symptoms and promoting PTG among BC patients (Hefferon et al., 2010; Trindade et al., 2018). Doctors and nurses should help BC patients by listening to their new bodies and using their bodies as a tool to promote PTG and their overall health.

Social support played a critical role in protecting psychological health, which was in line with previous studies (Hill and Watkins, 2017; Kugbey et al., 2020). Although most patients had high levels of social support in our study, social support for Chinese breast cancer patients was still insufficient compared with patients in Korea (Kim and Jang, 2019), Malaysia (Shao et al., 2020), and even in Ethiopia (Wondimagegnehu et al., 2019). So promoting social support for BC patients was necessary in China. Social support had a negative effect on depressive symptoms and had a positive effect on PTG based on our results. Cancer patients with higher levels of social support could be more effective when coping with stressful situations (Yeung and Lu, 2018) and relieving depressive symptoms (Kugbey et al., 2020). Social support had been considered a protective factor for physical and mental health among BC patients (Leung et al., 2016), especially family support (Wondimagegnehu et al., 2019). Family or friends could help cancer patients solve the problems through a positive pathway (Li et al., 2016), thereby resisting depressive symptoms and resulting in PTG among BC patients. Perceived support from partners, family members, or friends could positively foster PTG among BC patients (Romeo et al., 2019). Social support could also promote long-term PTG in cancer patients, even 8 years after cancer diagnosis (Schroevers et al., 2010). Emotional support could offer new perspectives on the traumatic event through both cognitive and emotional mechanisms (Cormio et al., 2017) and trigger PTG.

As expected, social support mediated relationships between body image and psychological outcomes. A higher level of body image disturbance might lead to a lower level of social support and result in a higher level of depressive symptoms and a lower level of PTG. Social support was a mediator in associations between body image and psychological outcomes (depressive symptoms and PTG). Our results suggested that we could prevent depressive symptoms and promote PTG in BC patients by enhancing the level of social support and decreasing the level of body image. Body image adjustment might be particularly associated with enough support from partners or intimate relationships (Scott et al., 2004). Melissant et al. (2019) also indicated that the importance of appearance was significantly associated with perceived support from the public media (Melissant et al., 2019). The result was also in line with Spatuzzi et al. (2016). Therefore, interventions based on facilitating and improving social support together with enhancing body image management should be implemented in enhancing psychological health among BC patients.

## Study limitations

This present study had several limitations. First, due to the cross-sectional design, our study could not assess the causal relationships among study variables. Longitudinal studies are thus warranted. Second, all patients were recruited from one major public hospital, which might potentially limit the generalization of our findings.

## Conclusion

Chinese BC patients suffered moderate or high levels of depressive symptoms and PTG. Body image was negatively associated with PTG and positively associated with depressive symptoms. Social support played an independent and protective role in promoting psychological health, and significantly mediated relationships between body image and psychological outcomes. Consequently, psychosocial intervention strategies integrating adequate social support and body image should be included in the prevention and treatment strategies of PTG and depressive symptoms among BC patients.

## Data availability statement

The original contributions presented in the study are included in the article/supplementary material, further inquiries can be directed to the corresponding author.

## Ethics statement

The study design was approved by the Committee on Human Experimentation of China Medical University (71904204). Informed consent was signed by each participant. The privacy of patients was protected throughout the entire research. All methods were carried out in accordance with relevant guidelines and regulations.

## Author contributions

ML conceptualized the research paper, performed the survey, conducted data analyses, interpreted the data, and compiled the manuscript.

## Funding

This work was supported by the National Natural Science Foundation of China (Grant Number: 71904204).

## Conflict of interest

The author declares that the research was conducted in the absence of any commercial or financial relationships that could be construed as a potential conflict of interest.

## Publisher's note

All claims expressed in this article are solely those of the authors and do not necessarily represent those of their affiliated organizations, or those of the publisher, the editors and the reviewers. Any product that may be evaluated in this article, or claim that may be made by its manufacturer, is not guaranteed or endorsed by the publisher.
